# The Effects of Wuniuzao Green Tea on Mice With High‐Fat Diet‐Induced Liver Steatosis

**DOI:** 10.1002/fsn3.70505

**Published:** 2025-07-03

**Authors:** Yiwei Yuan, Jiangcheng Ye, Mingxiu Gong, Yifan Zhang, Qianqian Xu, Jin Zhao

**Affiliations:** ^1^ College of Life Sciences, Institute of Food Nutrition and Quality Safety China Jiliang University Hangzhou Zhejiang China; ^2^ Key Laboratory of Specialty Agri‐Product Quality and Hazard Cintrolling Technology of Zhejiang Province China Jiliang University Hangzhou Zhejiang China

**Keywords:** AMPK pathway, intestinal microbiota, lipid metabolism, liver steatosis, Wuniuzao green tea

## Abstract

Wuniuzao is a famous Chinese tea with a history of more than 300 years. It is suitable for bulk production with excellent quality. Most current studies have focused on the cultivation, aroma compounds, and antioxidant capacity of Wuniuzao tea. However, the biological effects of Wuniuzao green tea water (WGT) extract on the prevention of high‐fat diet (HFD)‐induced liver steatosis and modulation of the intestinal microbiota have not yet been reported. This study was conducted to fill these gaps. Mice were divided into four groups and fed a normal diet, HFD, HFD + atorvastatin (10 mg/kg, positive control), and HFD + WGT (300 mg/kg), respectively. Nine‐week administration of WGT reduced body weight, lipid accumulation, and serum lipid levels in HFD‐fed mice. Liver function and serum glucose tolerance were improved, and hepatic steatosis was inhibited in the WGT group. Moreover, WGT treatment activated the AMPK signaling pathway, suggesting a mechanism for its effect on hepatic steatosis. WGT supplementation also increased intestinal microbiota diversity and modulated the ratio of Firmicutes to Bacteroidetes. WGT significantly alleviated HFD‐induced liver steatosis and modulated HFD‐induced intestinal microbial dysbiosis, suggesting its potential as a dietary supplement against HFD‐induced obesity and associated liver steatosis.

## Graphical abstract

WGT can significantly alleviate HFD‐induced liver steatosis, regulate intestinal microbial disorders, and serve as a dietary supplement to tackle obesity in mice.

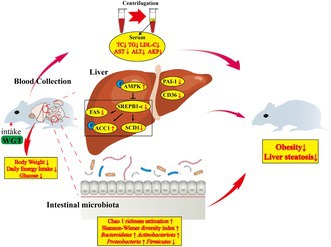

## 1 Introduction

Obesity is currently one of the most common public health issues. As a chronic metabolic disease (Upadhyay et al. 2018), obesity is a major risk factor for various complications such as diabetes (Sharma et al. 2020), cardiovascular disease (Kotsis et al. 2018), and non‐alcoholic fatty liver disease (Polyzos et al. 2019). Recently, the term metabolic dysfunction‐associated steatotic liver disease (MASLD) has been introduced to replace non‐alcoholic fatty liver disease (Rinella et al. 2023). Over the past few decades, clinical descriptions of obesity have largely focused on the analysis of body mass index (BMI) (WHO 2022). Fat accumulation resulting from energy imbalance is the primary characteristic of obesity, and reducing its risk primarily involves decreasing energy intake or increasing overall energy expenditure. However, long‐term adherence to healthy lifestyle changes, including diet and physical exercise, is often challenging for patients with obesity (Kushner 2018). Moreover, because synthetic drugs often have side effects (Kang and Park 2012), natural plant products are gaining popularity as dietary supplements for obesity management.

Tea, a natural, non‐toxic, safe product, is consumed as a beverage by more than two‐thirds of the global population. Green tea is reported to have health benefits: it helps attenuate or reverse hypercholesterolemia (Kang et al. 2021), hyperlipidaemia (Ma et al. 2019), and non‐alcoholic fatty liver (Labdi et al. 2018). The major bioactives in green tea include polyphenols, caffeine, and free amino acids. The most abundant and bioactive polyphenol in green tea is epigallocatechin‐3‐gallate (EGCG). Green tea extract was shown to alleviate the development of liver steatosis in high‐fat diet (HFD)‐fed mice (Karolczak et al. 2019). Moreover, green tea extract can improve hepatic metabolism by increasing adiponectin levels (Marinovic et al. 2022) and has been shown to prevent obesity and alleviate gut dysbiosis by improving intestinal barrier function and intestinal microbiota abundance (Dey et al. 2019).

Wuniuzao is a famous Chinese tea with a history of more than 300 years, and it originated in Yongjia County, Zhejiang Province. It germinates very early, exhibits good stress resistance, and produces high yields with excellent quality. However, only limited studies have assessed the anti‐obesity properties of Wuniuzao green tea. It remains unclear how Wuniuzao green tea affects lipid metabolism. Therefore, this study aimed to investigate the effects of Wuniuzao green tea (WGT) extract on HFD‐induced obesity and hepatic steatosis in mice, and to explore the underlying molecular mechanisms. The study assessed AMPK signaling pathway regulation, adipogenesis‐related gene expression in the liver, improvement of the intestinal microbiota composition, and the effects of WGT against obesity.

## 2 Materials and Methods

### 2.1 Preparation of WGT

Wuniuzao green tea was obtained from Sannong Tea Co. Ltd. (Yongjia County, Zhejiang, China) in April 2021. Following the method of Saklar et al. (2015), green tea (100 g) was extracted using hot water (1000 mL, 95°C) for 5 min. The aqueous extract was filtered and freeze‐dried at −55°C for 48 h. The obtained WGT was then stored in a freezer at −20°C until use.

### 2.2 Measurement of Total Polyphenol, Catechin, Caffeine, and Free Amino Acid Concentrations

The total polyphenol concentration in WGT was determined using spectrophotometry, while catechin and caffeine concentrations were determined using high‐performance liquid chromatography (HPLC). (ANSI Webstore 2022) Ninhydrin colorimetric analysis was used to quantify the free amino acid concentration (Moore and Stein 1954). Standard samples of EGCG, epicatechin gallate (ECG), epigallocatechin (EGC), epicatechin (EC), catechin, gallic acid (GA), and caffeine were acquired from Yuanye Biotechnology Co. Ltd. (Shanghai, China). The main chemical components of the WGT extract are displayed in Table 1.

**TABLE 1:** Chemical profile of Wuniuzao green tea.

Component	Content (%)
Total polyphenols	20.39 ± 0.45
Caffeine	3.78 ± 0.01
Free amino acid	6.82 ± 0.23
EGC	2.10 ± 0.20
C	0.87 ± 0.75
EC	1.04 ± 0.22
EGCG	6.18 ± 1.13
ECG	2.41 ± 0.02
Total catechin	12.59 ± 1.45

Abbreviations: C, catechin; EC, epicatechin; ECG, epicatechin gallate; EGC, epigallocatechin; EGCG, epigallocatechin gallate.

### 2.3 Animal Care and Treatment

All animal procedures and testing were performed according to the Experimental Animal Ethical Review Committee of China Jiliang University under the approval number (2023) 001. Eight‐week‐old male C57BL/6 mice (SYXK [Zhejiang] 2018‐0009) (*n* = 40) obtained from Hangzhou Ziyuan Experimental Animal Technology Co. Ltd. (Hangzhou, China) were placed in a conventional environment with unrestricted access to food and water. The mice were then randomly divided into four groups of 10 mice each following a week of acclimation feeding. The normal control (NC) food group was fed normal chow (Jiangsu Xietong Biological Engineering Co. Ltd.) and pure water. The HFD group, positive control (PC) group, and WGT group were fed an HFD containing 60% fat calories (D12492; Jiangsu Xietong Biological Engineering Co. Ltd.) and pure water. The WGT group was fed an HFD + WGT (dissolved in distilled water). According to previous studies (Dey et al. 2019; Zhu et al. 2020), WGT (300 mg/kg body weight) received Wuniuzao green tea aqueous extract per day. The dosage is equivalent to 1.5 cups of green tea consumed for an adult (70 kg) per day. Atorvastatin (10 mg/kg body weight) was administered by daily gavage. The mice in the other groups received the same amount of distilled water. Body weight was determined weekly (YP1002N; Techcomp Balance Instrument Co. Ltd., Shanghai, China). The mice were fasted for 12 h and sacrificed through carbon dioxide inhalation after 9 weeks of intervention. Serum, liver, and colonic feces samples were collected and stored at −80°C.

### 2.4 Oral Glucose Tolerance Test (OGTT)

During the fifth week of the experiment, 12 h‐fasted mice were subjected to an OGTT by oral glucose administration (2 g kg^−1^). Using an Accu‐Chek Performa glucometer and test strips (Roche, Basel, Switzerland), the glucose concentration in tail blood was measured at 0, 15, 30, 60, and 120 min after oral glucose administration.

### 2.5 Hematoxylin and Eosin (HE) Staining

The same part from the right lobe of the liver was taken during dissection and fixed in fixative (10% neutral buffered formalin) for 24 h. The fixed liver was then embedded in paraffin. The samples were sectioned for HE staining. Histopathological analysis was performed for each sample using HE staining.

### 2.6 Measurement of Metabolite Concentrations

The following serum and liver‐related marker concentrations were measured following the test kit instructions (Nanjing Jiancheng Biotechnology Co. Ltd.): including total triglycerides (TG), total cholesterol (TC), high‐density lipoprotein cholesterol (HDL‐C), low‐density lipoprotein cholesterol (LDL‐C), glutamic aminotransferase (AST), alanine aminotransferase (ALT), and alkaline phosphatase (ALP).

### 2.7 Real‐Time PCR

The TRIzol Plus RNA Purification Kit (Thermo Fisher, USA) was used to extract total RNA from the liver, and the RNase‐Free DNase Set (Qiagen, Shanghai) was used to eliminate DNA contaminants. The Nanodrop 2000 UV–Vis Spectrophotometer (Beckman, USA) was then used to spectrophotometrically measure the RNA quantity. SuperScript III First‐Strand Synthesis SuperMix (Thermo Fisher, USA) was used to create single‐stranded cDNA from total RNA, and Power SYBR Green PCR Master Mix was used to conduct real‐time PCR (Applied Biosystems). The PCR conditions were as follows: 95°C, 1 min; 40 cycles (95°C, 15 s, 63°C, 25 s, fluorescence collected), 55°C–95°C melting point curve. Glyceraldehyde‐3‐phosphate dehydrogenase (GAPDH) was set as an endogenous reference. The comparative cycle threshold (2^−ΔΔCt^) approach was employed to statistically examine the expression levels of each gene. The primer sequences are displayed in Table 2.

**TABLE 2:** Real‐time PCR primers and conditions.

Gene	Primer sequences (5′ to 3′)	Size (bp)	Annealing temperature (°C)
*GAPDH*	GAAGGTCGGTGTGAACGGATTTG	127	60
	CATGTAGACCATGTAGTTGAGGTCA		
*SCD1*	GCAAGCTCTACACCTGCCTCTTC	110	60
	CAGCCGTGCCTTGTAAGTTCTG		
*CD36*	CAGATGACGTGGCAAAGAACAG	217	60
	GAACCAAACTGAGGAATGGATCT		
*ACC1*	CGTGCAATCCGATTTGTTGTCATG	86	60
	GGAACATAGTGGTCTGCCATCT		
*PAI‐1*	GTGGCCAATGGAAGACTCCTT	130	60
	GTGGTGAACTCAGTGTAGTTGAACT		
*SREBP1c*	CCCGGCTATTCCGTGAACAT	111	60
	GCAGATATCCAAGGGCATCTGA		
*FAS*	GCTTTGCTGCCGTGTCCTTCTA	102	60
	CTGTCTTGGCACGCAGCAGT		

Abbreviations: ACC1, acetyl‐CoA carboxylase 1; CD36, cluster of differentiation 36; FAS, fatty acid synthetase; GAPDH, glyceraldehyde‐3‐phosphate dehydrogenase; PAI‐1, plasminogen activator inhibitor‐1; SCD1, stearoyl‐coenzyme A desaturase 1; SREBP1c, sterol regulatory element binding protein‐1c.

### 2.8 Immunoblot Analysis

RNA immunoprecipitation (RIPA) buffer (with a protease inhibitor cocktail) was used to extract the total protein from the liver; the extracted total protein was then quantified using a BCA quantification kit. An 8%–12% separator gel and 5% concentrator gel were prepared, and 60 μg of total protein per well was loaded for sampling (10–15 μL per well, 60 V for concentrator gel, and 80 V for separator gel, electrophoresis run‐time of approximately 2 h). The SDS‐PAGE gel was soaked in Tris‐glycine transfer buffer with 5% methanol for at least 5 min after 20 s of soaking the PVDF membrane in methanol. The membrane was transferred, immersed in T‐TBS (5% BSA), left at room temperature (1 h), and then washed thrice in T‐TBS for 5 min. The secondary antibody was added to the membrane and incubated for 1 h (room temperature) after the primary antibody was incubated overnight (4°C). Approximately 1 mL of the ECL working solution was prepared following the instructions, and SuperSignal West Dura Extended Duration Substrate was used. The X‐ray film was exposed for 5–10 min in a dark box for development and fixation after the transfer film was incubated for 1 min at room temperature. The excess ECL reagent was removed, and the film was wrapped with cling film. Using ImageJ 1.8.0 image processing software (USA), the optical density values of the bands were quantified thrice, and the relative expression of the target protein was expressed as follows: target protein (optical density value)/internal reference (optical density value) × 10^*n*^. The details for primary and secondary antibodies (dilution, manufacturer) have been provided in Table 3.

**TABLE 3:** Antibody information.

Antibody	Manufacturer	Dilution
Fas	Abcam	1: 3000
p‐ACC1	CST	1:1000
ACC1	CST	1:1000
SREBP1	Abcam	1:2000
p‐AMPKα	CST	1:1000
AMPKα	Abcam	1:2000
GAPDH	Abcam	1:10,000
Goat anti‐rabbit IgG (H + L)	Thermo	1:5000
Goat anti‐mouse IgG (H + L)	Thermo	1:5000

*Note:* Primary antibodies (Fas, p‐ACC1, ACC1, SREBP1, p‐AMPKα, AMPKα, GAPDH); secondary antibodies (goat anti‐rabbit IgG, goat anti‐mouse IgG).

### 2.9 16S rDNA Gene Sequences

Tsingke Technology Co. Ltd. was in charge of the 16sDNA sequencing (Beijing, China). Following total RNA extraction from the mouse colon fecal samples, the V3–V4 region was amplified using certain primers (338F: 5′‐ACTCCTACGGGAGGCAGCA‐3′, 806R: 5′‐GGACTACHVGGGTWTCTAAT‐3′). To create a sequence library, the products were purified, measured, and homogenized. The constructed libraries were initially submitted for library quality control, after which high‐throughput sequencing libraries that met the quality control standards were used (using Illumina NovaSeq 6000). Base Calling analysis was used to convert the raw picture data files from high‐throughput sequencing into sequenced reads. The double‐end sequence data collected from the high‐throughput sequencing were combined into one sequence tag based on the overlap between the reads. Using UCLUST (Edgar 2010) in QIIME (Caporaso et al. 2010), the tags were clustered into OTUs (97% similarity), and the OTUs were taxonomically labeled according to SILVA (for bacteria) taxonomic databases (Quast et al. 2012). Mothur 1.30 was used to assess the samples' alpha diversity indices. Using the QIIME program, a beta diversity study was conducted. Different samples' levels of species diversity were evaluated for similarities. To further analyze the characteristic intestinal microbiota and significant abundance difference characteristics in each group of mice, line discriminant analysis effect size (LEfSe) was used to discover and interpret biomarkers of statistical differences for high‐dimensional data between the groups. Moreover, the species composition obtained through taxonomic annotation of OTUs from high‐throughput sequencing were compared with the KEGG PATHWAY database (Kanehisa 2000) and functionally annotated at their compendial level. Welch's *t*‐test was used with STAMP (Parks et al. 2014) to compare the KEGG pathways between the groups.

### 2.10 Statistical Analysis

All data are expressed as means ± standard deviations (SD). Statistical differences were analyzed using one‐way ANOVA followed by the Duncan test using SPSS 20.0 software. *p* < 0.05 was considered statistically significant.

## 3 Results

### 3.1 WGT Protects Mice From Obesity Induced by HFD

**FIGURE 1:**
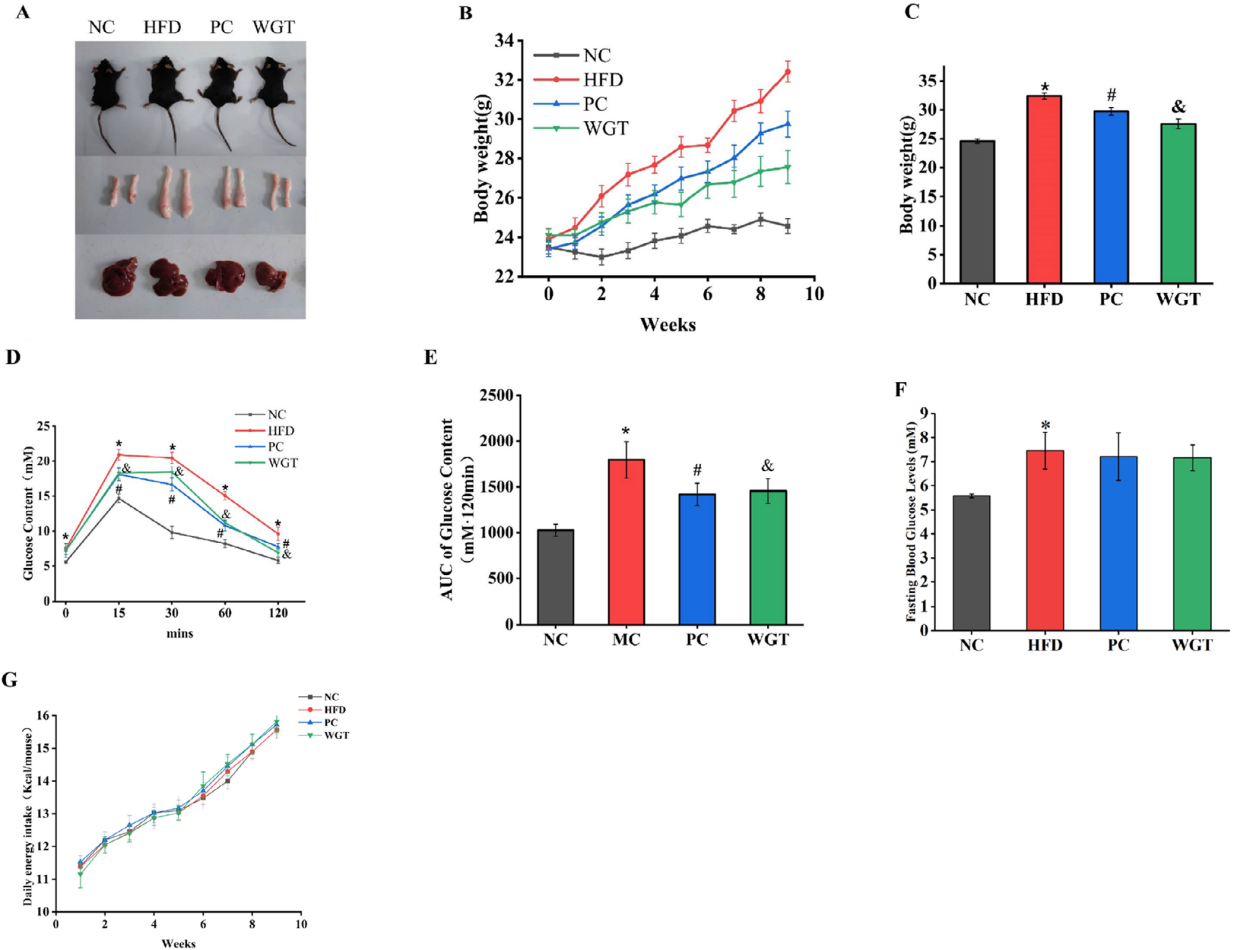
WGT supplementation prevents high‐fat diet‐induced obesity in mice. (A) Representative photos of a mouse, epididymal white adipose tissue and liver tissue in each group. (B) Body weight changes in each group. (C) Final body weight after the 9 week intervention in each group. (D) Blood glucose was determined at 0, 15, 30, 60, and 120 min after glucose administration. (E) The AUC value (area under the curve) for the glucose tolerance test. (F) Fasting blood glucose levels. (G) Daily calorie intake. *n* = 6. One‐way analysis of variance: *p* < 0.05. *p < 0.05, HFD compare with NC mice. ^#^ *p* < 0.05, PC compared with HFD mice. ^&^ *p* < 0.05, WGT compared with HFD mice.

Compared to the NC group, mice fed the HFD gained significantly more body weight over the 9‐week period. However, WGT supplementation effectively inhibited this HFD‐induced weight gain (*p* < 0.05) (Figure 1B,C). It is interesting to note that there was no significant difference in the daily energy intake of the mice between the groups (*p >* 0.05) (Figure 1G). Livers from the HFD group showed visible enlargement compared to the NC group, an effect that was inhibited by both atorvastatin and WGT supplementation (Figure 1A). Compared to the HFD group, epididymal fat accumulation was reduced in the PC and WGT groups. Furthermore, the TC, TG, LDL‐C, and ALP serum concentrations were lower in the WGT group (*p* < 0.05), showing a consistent trend with the PC group. The serum levels of HDL‐C did not significantly change in any group (*p* > 0.05). Moreover, compared to the HFD group, serum ALT and AST concentrations were significantly decreased in mice treated with WGT (*p* < 0.05) (Table 4). The above results preliminarily determined that the hepatic steatosis model established by HFD was successful and that the intervention effect of WGT was more obvious.

**TABLE 4:** Serum parameters of the mice administered WGT.

Items	NC	HFD	PC	WGT
TC (mmol/L)	3.54 ± 0.13	6.01 ± 0.14^a^	5.04 ± 0.90^b^	4.58 ± 0.14^c^
TG (mmol/L)	0.57 ± 0.07	0.99 ± 0.04^a^	0.65 ± 0.03^b^	0.70 ± 0.11^c^
LDL‐C (mmol/L)	0.20 ± 0.10	0.64 ± 0.10^a^	0.55 ± 0.05^b^	0.49 ± 0.04^c^
HDL‐C (mmol/L)	3.11 ± 0.53	2.60 ± 0.56	2.87 ± 0.35	2.80 ± 0.73
AST (U/L)	60.99 ± 2.27	101.18 ± 2.42^a^	69.47 ± 0.46^b^	68.42 ± 0.63^c^
ALT (U/L)	15.87 ± 0.30	32.17 ± 0.92^a^	23.03 ± 0.18^b^	24.83 ± 0.34^c^
ALP (U/L)	57.91 ± 1.35	143.79 ± 0.33^a^	86.47 ± 0.50^b^	74.38 ± 0.70^c^

*Note:* *n* = 6. One‐way analysis of variance: TC, TG, LDL‐C, AST, ALT, and ALP (*p* < 0.05), HDL‐C (*p* = 0.466).

Abbreviations: ALP, alkaline phosphatase; ALT, alanine aminotransferase; AST, glutamic aminotransferase; HDL‐C, high‐density lipoprotein cholesterol; LDL‐C, low‐density lipoprotein cholesterol; TC, total cholesterol; TG, total triglycerides.

^a^ *p* < 0.05, HFD compared with NC mice.

^b^ *p* < 0.05, PC compared with HFD mice.

^c^ *p* < 0.05, WGT compared with HFD mice.

Mice fed an HFD tend to have impaired glucose tolerance, as do patients with obesity. An OGTT was conducted in the fifth week to assess glucose tolerance. The initial blood glucose levels in the HFD group were considerably higher (*p* < 0.05). At 15, 30, 60, and 120 min after the oral administration of a 20% glucose solution, the blood glucose levels in the HFD group were different from those in the NC, PC, and WGT groups (*p* < 0.05) (Figure 1D). Compared with the single‐point glucose values, the area under the glucose tolerance curve provided a more comprehensive and clearer picture of the extent of glucose changes (Figure 1E). The AUC of the HFD group was significantly greater than that of the NC group (*p* < 0.05). In comparison with the HFD group, the WGT group's glucose levels and AUC were significantly lower throughout (*p* < 0.05). Simultaneously, atorvastatin significantly reduced the AUC of the PC group (*p* < 0.05).

### 3.2 WGT Inhibits Liver Steatosis by Activating the AMPK Pathway in the Liver

**FIGURE 2:**
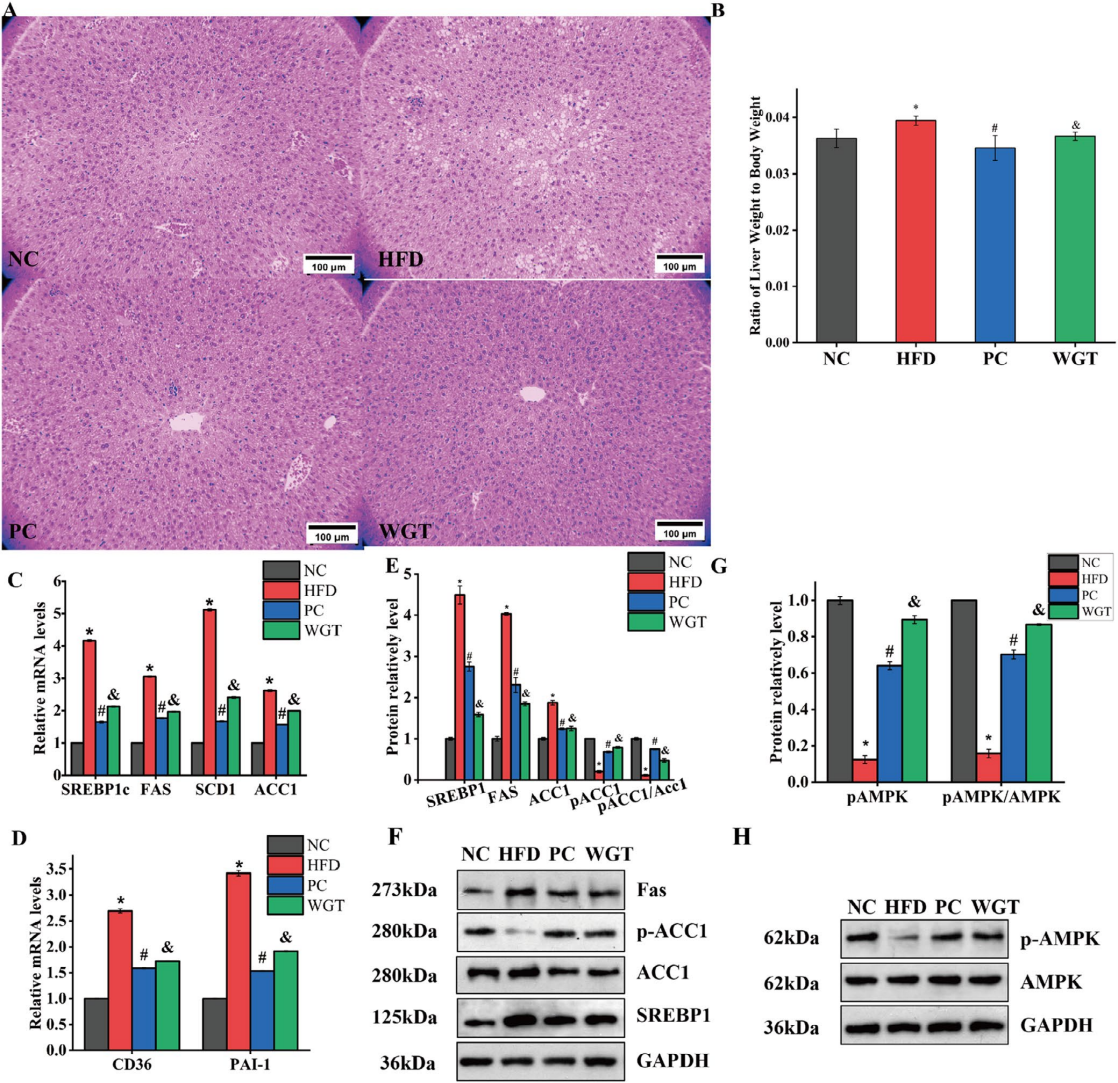
WGT reduces liver steatosis via activating the AMPK pathway. (A, B) Representative HE staining of liver (200×, scale bar = 100 μm), and the liver organ coefficient. (C, D) The transcriptional levels of SREBP1c, FAS, SCD1, ACC1, CD36 and PAI‐1. (E, F) The proteins expression levels of FAS, p‐ACC1, ACC1 and SREBP1. (G, H) The proteins expression levels of p‐AMPK and AMPK. *n* = 6. One‐way analysis of variance: *p* < 0.05. *p < 0.05, HFD compare with NC mice. ^#^ *p* < 0.05, PC compared with HFD mice. ^&^ *p* < 0.05, WGT compared with HFD mice.

**FIGURE 3:**
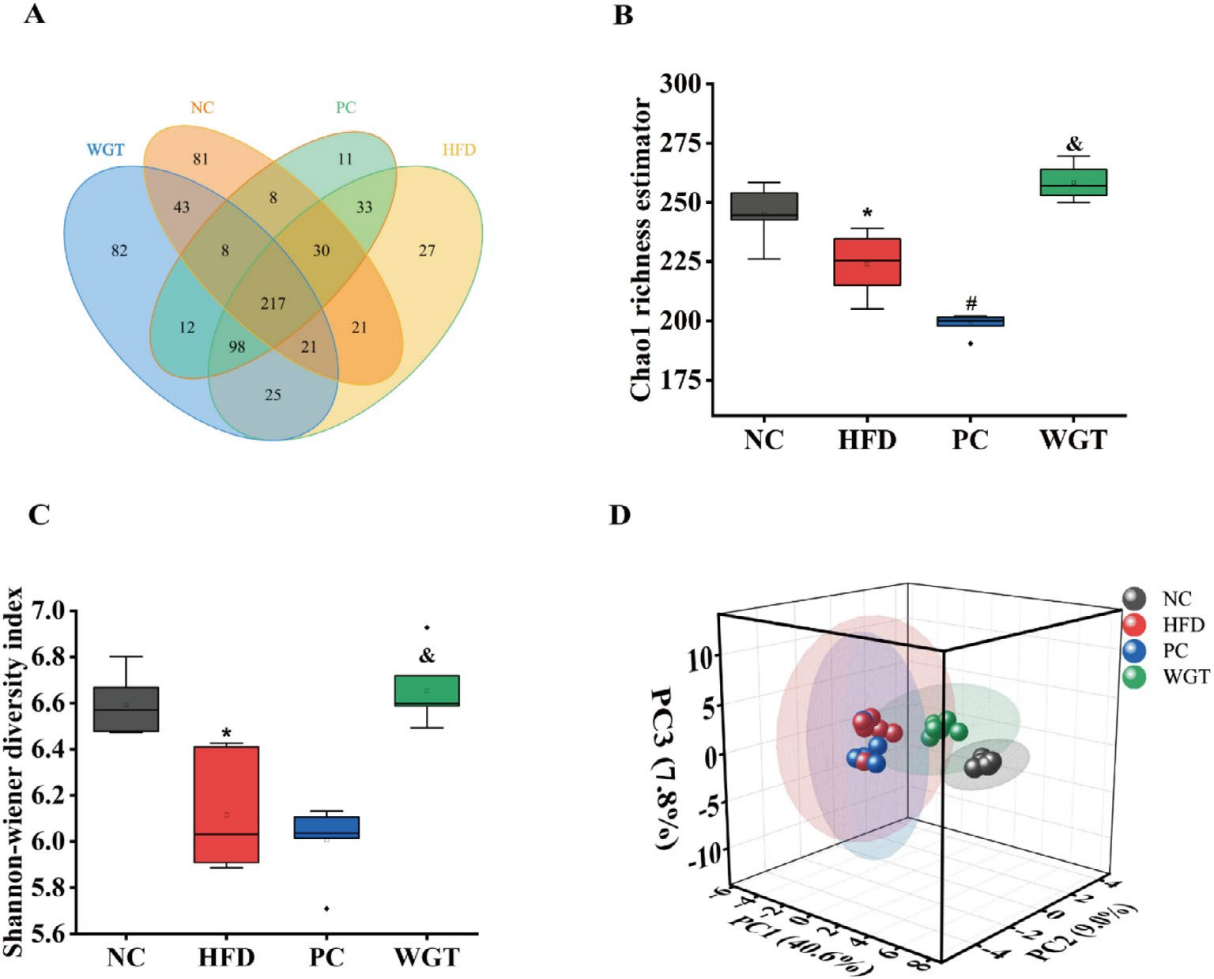
WGT influenced the diversity of intestinal microbiota in four groups of mice. (A) OTUs venn diagram. (B) Chao 1 richness estimation. (C) Shannon–Wiener diversity index. (D) PCA. One‐way analysis of variance: *N* = 6. *p* < 0.05. *p < 0.05, HFD compare with NC mice. ^#^ *p* < 0.05, PC compared with HFD mice. ^&^ *p* < 0.05, WGT compared with HFD mice.

As shown in Figure 2A, the results of HE staining of mice liver tissue sections indicated that the staining status of cells in liver sections of NC group mice was red‐blue with intact cell morphology. In contrast, hepatocytes in the HFD group were disorganized and exhibited extensive macrovesicular steatosis (large intracellular lipid droplets), along with some pyknotic nuclei. In the PC and WGT groups, hepatocyte organization was improved, and the size and number of intracellular lipid droplets were markedly reduced compared to the HFD group. Combined with the analysis in Figure 2B, the trend in liver weight of mice in each group was also consistent with the staining results. In this study, we examined the downstream genes of the adenosine 5′‐monophosphate (AMP)‐activated protein kinase (AMPK) metabolic pathway involving sterol regulatory element binding protein‐1c (SREBP‐1c), acetyl‐CoA carboxylase1 (ACC), fatty acid synthetase (FAS) and stearoyl‐CoA desaturase1 (SCD) (Figure 2C). SREBP1c, FAS, ACC1, and SCD1 transcript levels were also considerably upregulated in the HFD group (Figure 3C). Moreover, the relative mRNA expression levels of cluster of differentiation 36 (CD36) and plasminogen activator inhibitor‐1 (PAI‐1) were also examined (Figure 2D). Compared to the NC group, the expression of both CD36 and PAI‐1 was significantly increased in the HFD group (*p <* 0.05). Compared to the HFD group, WGT intervention significantly downregulated the expression of SREBP‐1c, FASN, ACC1, and SCD1, similar to the effect observed in the PC (atorvastatin) group (*p* < 0.05). CD36 is an important transmembrane glycoprotein whose main function is to promote long‐chain fatty acid transport, and the expression of CD36 was decreased in the liver tissues of the WGT and PC groups (*p* < 0.05). Furthermore, the transcript level of the PAI‐1 gene, which is related to metabolic syndrome and atherosclerosis formation, was reduced in the liver tissues of WGT mice (*p* < 0.05). WGT, with the lipid‐lowering and weight loss effects, could significantly regulate lipid metabolism‐related genes and then protect the liver tissue of mice induced by HFD. The results demonstrated that the liver tissue SREBP1, FAS, ACC1, and p‐ACC1 protein expression levels of mice in HFD were significantly increased (*p* < 0.05), which also indicated that WGT inhibited the growth in lipid regulation expression (Figure 2E–H). Compared to the HFD group, WGT treatment resulted in significantly lower protein levels of mSREBP‐1c and FASN, and significantly higher levels of ACC1 phosphorylation (p‐ACC1), leading to an increased p‐ACC1/ACC1 ratio (*p* < 0.05, Figure 2E–H). Total ACC1 levels were also reduced by WGT compared to the HFD group. Thus, WGT was successful in inhibiting the accumulation of fat via the AMPK pathway in the liver.

### 3.3 WGT Regulates the Intestinal Microbiota to Intervene in HFD‐Induced Liver Steatosis

A total of 1,919,446 reads were obtained from all four groups of 24 colonic stool samples sequenced with 16S rRNA in the V3–V4 region. The sum of 1,915,100 clean reads was produced after double‐ended reads were quality‐controlled and merged. A minimum of 79,387 clean reads with an average of 79,796 clean reads were produced in each sample. All clean reads were clustered using USEARCH 10.0 (97% similarity level), and 717 OTUs were obtained. A Venn diagram illustrates the distribution of OTUs among the four groups, showing 217 shared OTUs across all groups, as well as unique OTUs specific to each group (Figure 3A). This indicates considerable variation in the intestinal microbiota composition among the groups.

The HFD, atorvastatin, and WGT could significantly influence the composition of the intestinal microbiota in mice. Compared with the NC group, the alpha (α) diversity analysis based on Chao1 richness estimator and Shannon–Wiener diversity index revealed that the relative abundance of intestinal microbiota was significantly lower in the HFD group (*p* < 0.05) (Figure 3B). Then, Chao1 richness estimator was considerably greater in the WGT group (*p* < 0.05) and the PC group was downregulated (*p* < 0.05). Meanwhile, in the PC group, the Shannon–Wiener diversity index was similar to that of the HFD group, while it increased in the WGT group (*p* < 0.05), which revealed the diversity of intestinal microbiota can be improved by supplementation with WGT. Beta (β) diversity analysis using principal component analysis (PCA) showed clear separation in the microbial community structure between the groups (Figure 3D). The first three principal components (PC1, PC2, and PC3) accounted for 40.6%, 9.0%, and 7.8% of the total variance, respectively. The overall distribution of the HFD group was different from the others. We also found a more dispersed distribution of intestinal microbiota in the PC group, which was very similar to that of the HFD group. The WGT group was closer to the NC, and its intestinal microbiota moved towards the NC group in the *x*‐axis direction, which showed the intestinal microbiota of mice have a normalizing trend under the supplementation of WGT.

**FIGURE 4:**
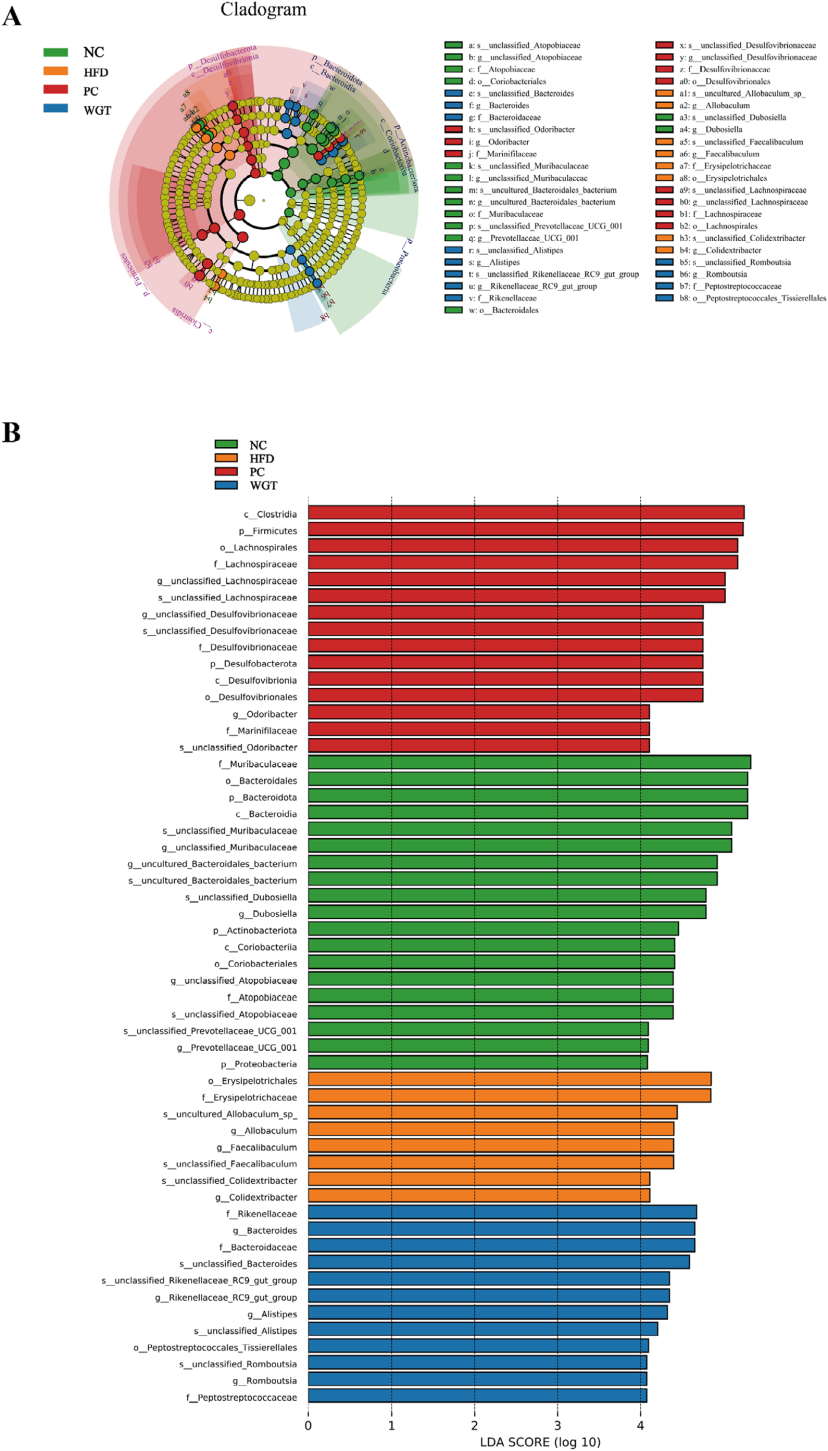
Intergroup LEfSe analysis of gut microbiota in each group. (A) Evolutionary branching in each group. (B) LDA values in each group. *n* = 6.

The study has performed LEfSe (difference analysis [LDA score > 4]) based on the intestinal microbiota of each group of mice (Figure 4). The different circles show various taxonomic levels. Within the same taxonomic level, the size of each small circle represents a taxonomic unit that is proportional to the relative abundance. The species with no tremendous contrasts will be shaded consistently in yellow, as well as the other differences that will be colored by the group with the highest species abundance. Different subgroups are represented by different colors. Furthermore, nodes represented by different colors mean the intestinal microbiota plays an essential role in the color‐represented subgroup. The histogram provides the distribution of LDA values for each group of samples from different species levels (Figure 4B). The LDA scores of species are represented by horizontal coordinates, and species with LDA values > 4 are statistically different intestinal microbiota. Combined with the analysis in Figure 4A,B, 19 intestinal microbiotas from different levels were detected with significant differences in the NC group. The community structure of intestinal microbiota was influenced by Muribaculaceae, Bacteroidales, Bacteroidota, and Bacteroidia. Six intestinal microbiotas from different levels were identified in the mice fed with HFD, exhibiting significant differences. Similarly, intestinal microbiota such as Erysipelotrichales, Erysipelotrichaceae, unclassified *Allobaculum* sp., and *Allobaculum* affect the community structure of intestinal microbiota. Fifteen intestinal microbiotas from different levels were detected to have significant differences in the PC group, and intestinal microbiota such as Clostridia, Firmicutes, Lachnospirales, and Lachnospiraceae had a great effect on the community structure of intestinal microbiota. The experimental results can be observed with twelve intestinal microbiotas in the WGT group, such as Rikenellaceae, Bacteroides, Bacteroidaceae, and unclassified Bacteroides, which had a great influence on the community structure of the intestinal microbiota.

**FIGURE 5:**
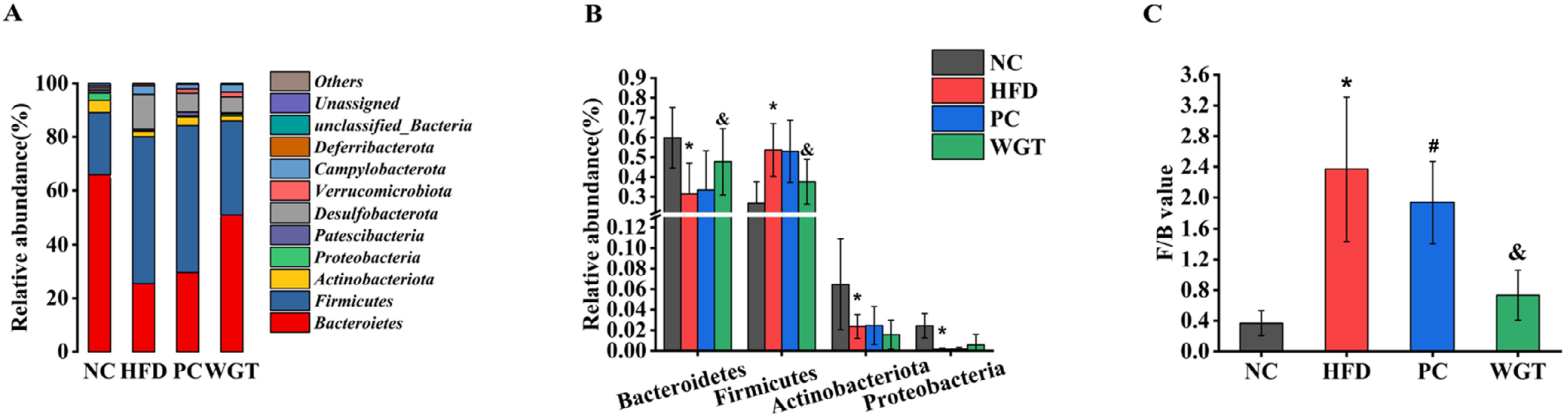
Effect of relative abundance of intestinal microbiota in four groups of mice. (A) Relative abundance of intestinal microbiota at the Phylum level. (B) The trend of changes in the four intestinal microbiotas. (C) The ratio of Firmicutes to Bacteroidetes (F/B). *n* = 6. One‐way analysis of variance: *p* < 0.05. *p < 0.05, HFD compare with NC mice. ^#^ *p* < 0.05, PC compared with HFD mice. ^&^ *p* < 0.05, WGT compared with HFD mice.

The relative abundance of major bacterial phyla was analyzed (Figure 5A). Significant differences were observed among the four groups at the phylum level, particularly for Bacteroidetes, Firmicutes, Actinobacteriota, and Proteobacteria. The test results showed that the relative abundance of Bacteroidetes, Actinobacteriota, and Proteobacteria was reduced by HFD (*p* < 0.05), while Firmicutes increased (*p* < 0.05) (Figure 5A). In addition to the predominant microbiota in the NC group, a clear expansion in the relative abundance of Desulfobacterota and Compylobacterota was discovered. Furthermore, the changes in intestinal microbiota prompted by HFD have not interfered with atorvastatin. Figure 5B demonstrates that the relative abundance of Firmicutes in the HFD group decreased with WGT (*p* < 0.05); at the same time, the abundance of Bacteroidetes decreased (*p* < 0.05). The ratio of Firmicutes to Bacteroidetes (F/B), often considered an indicator of gut dysbiosis, was significantly increased in the HFD group compared to the NC group. WGT supplementation significantly reduced this ratio compared to the HFD group (*p <* 0.05).

**FIGURE 6:**
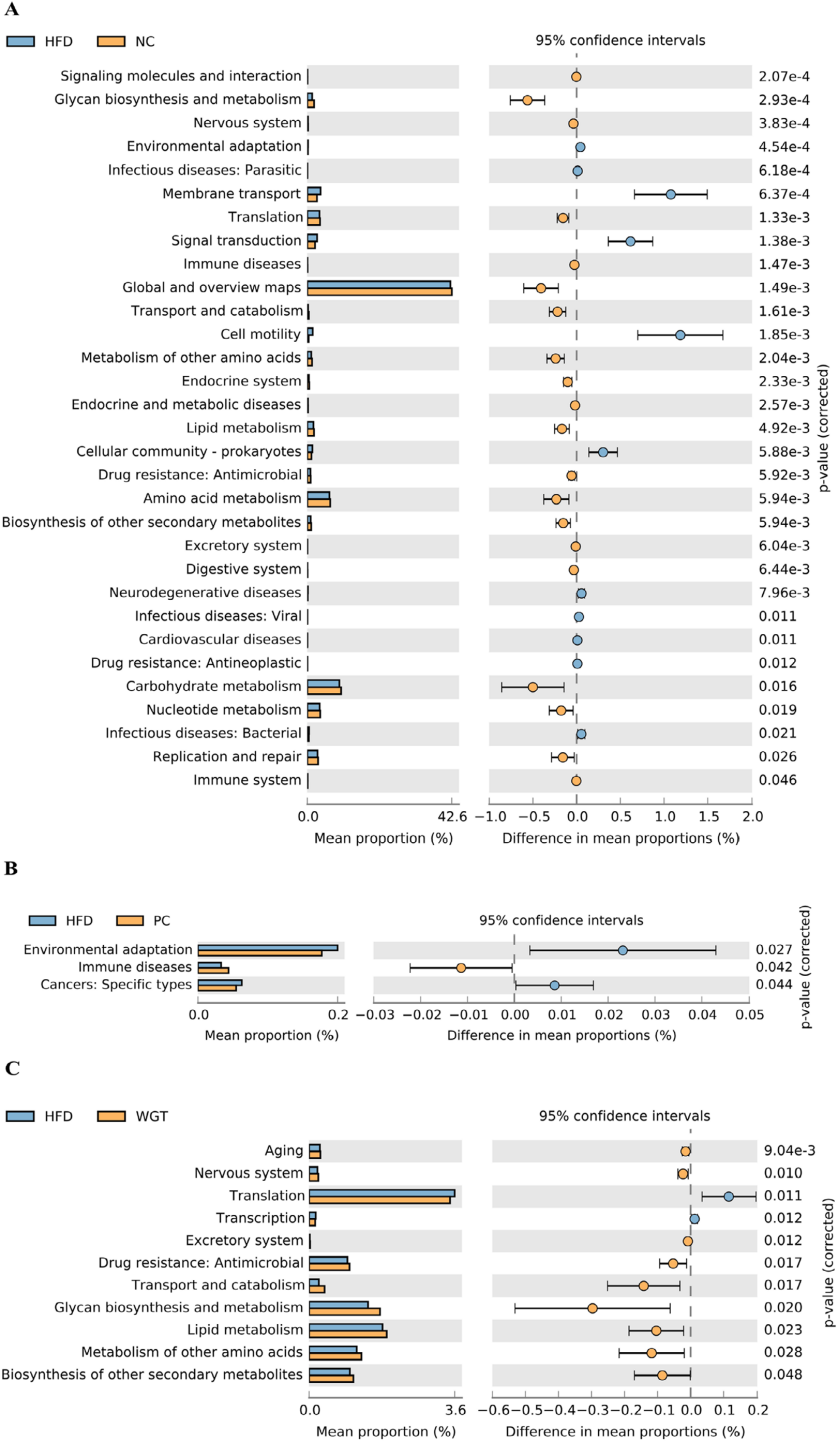
The functional abundance of intestinal microbiota differed significantly between different groups of mice. (A) The functional abundance in HFD group. (B) The functional abundance in PC group. (C) The functional abundance in WGT group. *n* = 6.

The intestinal microbiota leads to changes in the functional abundance of metabolic pathways in the mice (Figure 6). STAMP differential analysis (Welch's *t*‐test) comparing the HFD group to the NC group identified 31 KEGG pathways with significantly altered abundance (*p* < 0.05, Figure 6A). Of these, 11 pathways were significantly increased, and 20 were significantly decreased in the HFD group. These altered pathways were primarily related to lipid metabolism, glycan biosynthesis and metabolism, nucleotide metabolism, and amino acid metabolism. Atorvastatin altered only three functional abundances of environmental adaptation, immune diseases, and cancers: specific types in the intestinal microbiota of obese mice. A total of 11 functional abundances were changed in the HFD mice under WGT intervention. The abundance of nine functions was enhanced, including glycan biosynthesis and metabolism, lipid metabolism, metabolism of other amino acids, and biosynthesis of other secondary metabolites, while translation and transcription functions were reduced.

In summary, WGT regulates the intestinal microbiota to prevent hepatic steatosis, which is caused by HFD. WGT is closely related to the intestinal metabolic activity of the host and has a significant effect on the abundance and diversity of the intestinal microbiota of obese mice. The active substances in WGT may be metabolized by the intestinal microbiota, change the environment of the microbiota in the digestive system, and affect the biological function of the microbiota. Suppressing obesity can be achieved by regulating the abundance of the microbiome, which is related to the metabolic function of the host.

## 4 Discussion

Recently, the habit of drinking tea after meals in nomadic people, which mitigates the negative effects of high‐calorie intakes and HFD, has attracted widespread attention. Therefore, it is necessary to explore the molecular mechanisms underlying hepatic steatosis in obese mice fed an HFD. The results revealed that obese mice exhibited hyperlipidemia. These mice showed increased body weight and blood glucose levels. Glucose tolerance was significantly impaired, and liver hypertrophy with steatosis and abnormally elevated lipid levels was observed. These findings are similar to those reported by a previous study (He et al. 2020). These results demonstrated that the hepatic steatosis model using C57BL/6 mice was successfully established.

In this study, the intervention effect of WGT was found to be consistent with that of atorvastatin. Compared with the HFD group, body weight was significantly reduced in the WGT and PC groups. Glucose tolerance is an important indicator and is closely related to type 2 diabetes mellitus. It has a pathophysiological link with hepatic steatosis (Fan et al. 2022; Li, Fang, et al. 2020; Kim et al. 2019). Fasting blood glucose levels and AUC also demonstrated the positive effects of WGT on glucose tolerance in mice fed an HFD. Substantial research has been conducted on the preventive benefits of green tea extracts on serum lipid levels (Huang et al. 2022; Zhou et al. 2022). WGT exhibits considerable activity in reducing serum lipid levels. LDL‐C is known as “bad cholesterol” and is responsible for transporting synthesized cholesterol to other tissues throughout the body. In contrast to LDL‐C, HDL‐C is responsible for transferring cholesterol from the peripheral tissues back to the liver for catabolism. The concentrations of LDL‐C and HDL‐C are linked to many complications of obesity (atherosclerosis, cardiovascular disease, and coronary heart disease) (Ueda et al. 2018; Hao and Friedman 2014; Hao et al. 2017). Our results demonstrated that WGT intervention markedly alleviated hyperlipidemia and hepatic steatosis in obese mice. The indicators of serum (AST and ALT), which reflect liver function (Mou et al. 2019; Pezeshki et al. 2016), were decreased in the WGT group, indicating that WGT could improve liver function in obese mice.

SREBP1c is a transcription factor belonging to the SREBP family and is highly expressed in the liver and adipose tissue (Ferré et al. 2021). It profoundly affects hepatic lipid metabolism in vivo because of its capacity to activate genes, including *ACC1*, *FAS*, and *SCD1*. CD36, a fatty acid transporter enzyme in the liver, is linked to pathological conditions (visceral obesity, insulin resistance, and non‐alcoholic fatty liver disease [NAFLD]) (Rada et al. 2020; Cifarelli et al. 2021). Studies have shown that the expression of hepatic de novo lipogenesis (DNL) genes (*FAS*, *ACC*, and *SCD1*) and their master regulator SREBP‐1c is downregulated by green tea extract supplementation (Santamarina et al. 2015; Bae et al. 2018; Li et al. 2016). Moreover, PAI‐1 promotes the growth of adipose tissue inflammation and exacerbates metabolic disorders in obese individuals (Liu et al. 2019; Wang et al. 2018; Zhou et al. 2021). In this study, the transcript levels of lipid metabolism‐related genes (*SREBP1c*, *FAS*, *ACC1*, *SCD1*, *CD36*, and *PAI‐1*) were markedly increased in the HFD group. Additionally, the immunoblotting analysis of hepatic DNL‐related protein expression levels (SREBP1, FAS, ACC1, and p‐ACC1) revealed trends consistent with their corresponding mRNA transcript levels. The results showed increased protein expression of SREBP1, ACC1, and FAS, and decreased protein expression of p‐ACC1, leading to a reduced relative ratio of p‐ACC1/ACC1. This indicated that HFD enhances hepatic lipid production.

As a switch for energy metabolism, AMPK is a key factor in regulating lipid metabolism. EGCG in green tea activates AMPK phosphorylation via a peptide hormone (leptin, lipocalin, etc.) mediated AMP non‐dependent pathways or AMPK upstream kinases (Santamarina et al. 2015). Hawk tea extract could increase AMPK and ACC phosphorylation levels and downregulate SREBP1c and FAS expression to prevent obesity (Tao et al. 2022). The results showed that the PC and WGT groups had higher levels of phosphorylated AMPK (p‐AMPK). In the WGT group, the transcript levels of *CD36* and *PAI‐1* were significantly lower, potentially lessening the likelihood of metabolic problems developing in obese mice. Additionally, SREBP1c, FAS, and ACC1 protein expression levels as well as mRNA transcript levels were significantly reduced in the livers of the WGT group. This was accompanied by increased ACC1 phosphorylation (higher p‐ACC1 levels), which prevents fatty acid production and reduces lipid accumulation in liver tissue. The study revealed that WGT had a significant alleviating effect on hepatic lipid accumulation in obese mice by regulating the AMPK signaling pathway.

This study investigated the effect of WGT intervention on intestinal microbiota in mice fed an HFD. The relationship between intestinal microbiota and liver steatosis has recently become a significant focus of research (Wang et al. 2020; Liu, Wang, et al. 2022). Various teas and their active ingredients affect hepatic lipid metabolic pathways by regulating the intestinal microbiota of obese mice (Ye et al. 2021; Lu et al. 2019; Chen et al. 2018; Liu, Chen, et al. 2022). It was found that WGT prevented the HFD‐induced decrease in intestinal microbiota diversity and abundance, whereas the PC group exhibited a similar trend to the HFD group. Bacteroidetes, Firmicutes, Actinobacteriota, and Proteobacteria phyla comprise the majority of bacteria in the human gut microbiota. The most abundant microbiotas are Bacteroidetes and Firmicutes, which account for about 90% of the intestinal microbiota (Gerritsen et al. 2011). Erysipelotrichales and Erysipelotrichaceae, Gram‐positive bacteria belonging to Firmicutes, were significantly altered in the HFD group, as shown in the results of LEfSe analysis. Carter et al. (2021) found overgrowth of Erysipelotrichales by characterizing the intestinal microbiota of mice in a model of non‐alcoholic fatty liver disease (NAFLD). The significant changes in *Allobaculum* associated with HFD are also matched with the research of others (Kong et al. 2019). Muribaculaceae, a negatively beneficial bacterium, was enriched in mice of the NC group (Guo et al. 2021). Clostridia is one of the key intestinal microbiotas associated with liver steatosis, which may affect CD36 expression and lipid absorption (Hong et al. 2021; Petersen et al. 2019). The Clostridia taxa belong to Firmicutes, and many Clostridia taxa can produce secondary bile acids (BAs) (Ridlon et al. 2016). In advanced hepatic steatosis, HFD could increase the levels of secondary BAs and Clostridia in the colon (Zeng et al. 2020). Lachnospirales and Rikenellaceae both showed alterations in the intestinal microbiota of obese mice (Liu, Cao, et al. 2022; Lv et al. 2020).

A dynamic balance within the intestinal microbiota is maintained in the gut of healthy hosts. Because of their association with the onset of obesity, the two major bacteria—Bacteroides and Firmicutes—have been referred to as considered a key indicator related to obesity (Prior et al. 2010). Weight gain, intestinal inflammation, and liver steatosis are directly related to the relative abundance of, or the balance between, these two phyla. Firmicutes can induce the development of hepatic steatosis by modulating fatty acid synthesis and lipogenesis (Chen et al. 2019; Li, Yan, et al. 2020). Similarly, F/B has been used as a biomarker of gut dysbiosis in NAFLD mice (Jasirwan et al. 2021; Lee et al. 2021; Gómez‐Zorita et al. 2019). The F/B ratio was abnormally increased in the HFD group, which led to an imbalance in host energy metabolism and caused an increased incidence of liver steatosis (Turnbaugh et al. 2008). At the phylum level, the abundance of the intestinal microbiota of mice in the WGT group differed from that of the HFD group, but no consistent trend was found in the PC group. The important changes in the abundance of Bacteroidetes and Firmicutes caused by HFD were counteracted with WGT supplementation. These suggest that WGT may alleviate hepatic steatosis and intestinal dysbiosis in obese mice. Alterations in the predicted functional abundance related to lipid metabolism, nucleotide metabolism, and amino acid metabolism were also consistent with the findings of Zhou et al. (2019). Zhang et al. (2018) also found that EGCG exerts anti‐obesity and anti‐hepatic steatosis effects by mediating beneficial bacterial populations and affecting metabolic pathways. In contrast, atorvastatin did not show a similar significant influence on the functional abundance of intestinal microbiota in mice. Further analysis suggested that atorvastatin did not counteract the disorder of intestinal microbiota caused by HFD, and its weight loss and lipid‐lowering properties may not be achieved primarily through regulating intestinal microbiota.

## 5 Conclusions

The results confirmed that WGT could significantly reduce body weight, hepatic lipid accumulation, and serum lipid concentrations, and improve glucose tolerance in obese mice. Thus, WGT supplementation could inhibit hepatic steatosis via the AMPK pathway in mice. Moreover, the ratio of Firmicutes to Bacteroidetes significantly decreased, and the abundance of the intestinal microbiota improved. These alterations in the gut microbiota may contribute to the modulation of lipid metabolic pathways, leading to obesity mitigation and hepatic steatosis alleviation in mice.

## Author Contributions

**Yiwei Yuan:** conceptualization (equal), data curation (equal), formal analysis (equal), investigation (equal), resources (equal), validation (equal). **Jiangcheng Ye:** conceptualization (equal), software (equal), visualization (equal), writing – original draft (equal), writing – review and editing (equal). **Mingxiu Gong:** data curation (equal). **Yifan Zhang:** data curation (equal). **Qianqian Xu:** visualization (equal), writing – review and editing (equal). **Jin Zhao:** conceptualization (equal), funding acquisition (lead), methodology (equal), project administration (equal), supervision (equal), writing – review and editing (equal).

## Ethics Statement

All experimental protocols involving animals were approved by the Animal Care and Welfare Committee of Life Science College and the Scientific Ethical Committee of China Jiliang University (No. CJLU2023001; 6 January 2023; Hangzhou, China).

## Consent

The authors have nothing to report.

## Conflicts of Interest

The authors declare no conflicts of interest.

## Data Availability

Data are contained within the article. The high‐throughput sequencing of 16S rRNA described here is accessible via NCBI accession number PRJNA903654. https://www.ncbi.nlm.nih.gov/search/all/?term=PRJNA903654.
